# A Novel Calpain Inhibitor Compound Has Protective Effects on a Zebrafish Model of Spinocerebellar Ataxia Type 3

**DOI:** 10.3390/cells10102592

**Published:** 2021-09-29

**Authors:** Katherine J. Robinson, Kristy Yuan, Stuart K. Plenderleith, Maxinne Watchon, Angela S. Laird

**Affiliations:** Neurodegeneration Treatment Team, Centre for Motor Neuron Disease Research, Faculty of Medicine, Health and Human Sciences, Macquarie Medical School, Macquarie University, Sydney 2109, Australia; katherine.j.robinson@mq.edu.au (K.J.R.); kristy.yuan@mq.edu.au (K.Y.); stuart.plenderleith@mq.edu.au (S.K.P.); maxinne.watchon@mq.edu.au (M.W.)

**Keywords:** calpain, spinocerebellar ataxia-3, Machado Joseph disease, polyglutamine, zebrafish

## Abstract

Spinocerebellar ataxia type 3 (SCA3) is a hereditary ataxia caused by inheritance of a mutated form of the human *ATXN3* gene containing an expanded CAG repeat region, encoding a human ataxin-3 protein with a long polyglutamine (polyQ) repeat region. Previous studies have demonstrated that ataxin-3 containing a long polyQ length is highly aggregation prone. Cleavage of the ataxin-3 protein by calpain proteases has been demonstrated to be enhanced in SCA3 models, leading to an increase in the aggregation propensity of the protein. Here, we tested the therapeutic potential of a novel calpain inhibitor BLD-2736 for the treatment of SCA3 by testing its efficacy on a transgenic zebrafish model of SCA3. We found that treatment with BLD-2736 from 1 to 6 days post-fertilisation (dpf) improves the swimming of SCA3 zebrafish larvae and decreases the presence of insoluble protein aggregates. Furthermore, delaying the commencement of treatment with BLD-2736, until a timepoint when protein aggregates were already known to be present in the zebrafish larvae, was still successful at removing enhanced green fluorescent protein (EGFP) fused-ataxin-3 aggregates and improving the zebrafish swimming. Finally, we demonstrate that treatment with BLD-2736 increased the synthesis of LC3II, increasing the activity of the autophagy protein quality control pathway. Together, these findings suggest that BLD-2736 warrants further investigation as a treatment for SCA3 and related neurodegenerative diseases.

## 1. Introduction

Spinocerebellar ataxia type 3 (SCA3), also known as Machado Joseph disease (MJD), is an inherited disease characterized by progressive motor dysfunction and degeneration of neurons, producing impaired balance and motor coordination, and leading to wheelchair dependence [1]. SCA3 accounts for 21–28% of all autosomal dominant spinocerebellar ataxias [2,3,4], with a high prevalence in areas such as the Azores of Portugal [5], Southeastern China [6], and north east Arnhem Land in Northern Australia [7]. SCA3 is the most prevalent form of spinocerebellar ataxia found globally [8]. SCA3 is caused by inheritance of an abnormal repeat expansion (CAG) mutation in chromosome 12 of the *ATXN3* gene [9,10]. Whilst the wild-type form of the *ATXN3* gene typically contains 12–44 CAG repeats, inheritance of *ATXN3* with 44 or more CAG repeats has been found to produce disease [5,11,12]. Repeat expansion length has been found to correlate with disease severity in SCA3 patients, with inheritance of longer repeat expansions resulting in earlier onset of disease, greater disease severity, and earlier mortality [5,11,12,13]. Despite the comprehensive understanding of the genetic cause of SCA3, there is a distinct lack of treatment options available to slow or halt SCA3 progression.

The *ATXN3* gene encodes the protein ataxin-3, with the CAG repeat region encoding a long string of glutamine (Q) amino acids, known as a polyQ region. The endogenous function of ataxin-3 has not been fully elucidated, however ataxin-3 is known to act as a de-ubiquinating enzyme [1] and to play a functional role in transcriptional processes [14,15,16]. Expression of ataxin-3 containing 44 or more polyQ repeats has been reported to produce some toxic effects whereby mutant, expanded ataxin-3 can bind with other essential proteins or receptors, causing dysfunction of these proteins or receptors [16,17,18]. Additionally, mutant ataxin-3 has been reported to form protein inclusions or aggregates in the brain of SCA3 patients [19,20,21,22]. The formation of these ataxin-3 protein aggregates and inclusions is hypothesized to play a role in the neuronal dysfunction and degeneration that occurs in SCA3 [16,19,22,23]. In 2004, Goti et al. [21] extracted the proteins present within ataxin-3 aggregates and confirmed the presence of full-length ataxin-3 protein, but also smaller ataxin-3 protein fragments containing the polyQ region. It has been demonstrated that protein fragments containing long polyQ regions (without surrounding protein sequences) are highly aggregation-prone, likely due to the polyQ tract folding and binding with itself, resulting in the formation of neurotoxic protein aggregates [16,24,25,26,27]. Findings from in vivo models expressing polyQ protein fragments, such as *Caenorhabditis elegans*, *Drosophila melanogaster*, and *Danio rerio* (zebrafish), have provided further evidence of this, with increased polyQ length resulting in greater neurotoxicity in vivo [28,29,30,31,32]. This mechanism, termed the ‘toxic fragment hypothesis’ has been proposed to play a role in a range of neurodegenerative diseases [33,34,35].

It is known that these ataxin-3 fragments are formed through proteolytic cleavage of the full-length ataxin-3 protein by protease enzymes, such as the calpain proteases [16,24,36,37,38]. Interestingly, the calpain proteases are activated by intracellular Ca^2+^ and increases in intracellular Ca^2+^ have been proposed to play a role in the pathogenesis of SCA3 [17,38,39]. Indeed, experimental evidence suggests that the activity of calpain enzymes may be increased in cells obtained from SCA3 patients [38,40], a phenotype that can be ameliorated via the genetic silencing of calpain-1 [40] or worsened via genetic silencing of calpastatin, an endogenous calpain inhibitor [37]. Furthermore, previous studies have demonstrated that calpain inhibitor compounds are capable of inhibiting the formation of ataxin-3 fragments in vitro [38,41]. It is therefore hypothesized that cleavage of ataxin-3 is primarily performed by the calcium dependent protease, calpain [37,42]. For this reason, various groups have recently investigated whether inhibiting ataxin-3 cleavage through calpain inhibition could be beneficial as a SCA3 treatment [38,41,43,44]. Studies demonstrating the protective effect of calpain inhibition for SCA3 have included rodent studies overexpressing calpastatin [42], genetic ablation of calpain-1 [40], and treatment of in vitro and in vivo models with calpain inhibitor compounds such as calpeptin, ALLN, and BDA-410 [38,41,43,44,45].

We previously found that treating our transgenic zebrafish model of SCA3 with the calpain inhibitor calpeptin leads to improved motor behavior (swimming distance) in an autophagy dependent manner [43]. We found that treatment with calpeptin resulted in decreased abundance of, not only the ataxin-3 cleavage fragments, but also the full-length ataxin-3 protein. Our study, and others, have demonstrated that calpain inhibition can produce a robust induction of autophagy [43,46,47,48,49,50], which may contribute to the therapeutic potential of calpain inhibitor compounds. Therefore, additional studies are required to confirm whether the beneficial effect of calpain inhibition seen in these studies is indeed inhibition of ataxin-3 cleavage, or perhaps another effect of calpain inhibition, such as induction of autophagy, leading to active degradation of toxic protein aggregates.

Here, we report the beneficial effect of a novel calpain inhibitor compound, BLD-2736, an inhibitor of calpain-1, 2, and 9 and cathepsin K. BLD-2736 is a highly water-soluble compound, with potency at low concentrations (IC50 in nM range for cell culture studies). We report that BLD-2736 improves the swimming of the SCA3 zebrafish and decreases the presence of ataxin-3 protein aggregates and the presence of full-length polyQ expanded human ataxin-3 protein in transgenic zebrafish. Together these findings suggest that BLD-2736 warrants further investigation for the treatment of SCA3.

## 2. Materials and Methods

### 2.1. Transgenic SCA3 Zebrafish

The present study utilized a previously described zebrafish model of SCA3 [43,45,51]. The transgenic zebrafish were produced through the crossing of zebrafish of a driver line expressing Gal4 VP16 driven by a HuC (elav) neuronal promoter [52] and a responder line zebrafish containing a bidirectional UAS driving dsRED and pEGFP-C1-ATXN3–84CAG expression. The transgenic zebrafish hence expressed both dsRED and EGFP-fused ATXN3. For the present study, driver and responder lines were crossed to generate F1 HuC-EGFP ataxin-3 lines, which were in-crossed to generate F2 embryos. *ATXN3* plasmids were a kind gift from Henry Paulson (Addgene, plasmid no. 22123, Watertown, MA, USA [53]). All animal experiments were performed in accordance with the Code and approved by the Animal Ethics Committee of Macquarie University (2016/004). Zebrafish were housed in a standard recirculating aquarium system maintained at 28.5 °C. Zebrafish embryos aged 1 day post fertilization (dpf) were screened for fluorescence (EGFP and dsRED), indicating expression of transgenic lines. Embryos were dechorionated and housed in 6-well plates (12–25 larvae per well).

### 2.2. Calpain Inhibitor Testing

In the present study, the therapeutic benefit of two calpain inhibitor compounds was tested. A novel inhibitor of calpain-1, 2, and 9 and cathepsin K, BLD-2673, was provided by Blade Therapeutics. Calpeptin, a calpain-1 and 2 inhibitor, was purchased from Sapphire Bioscience (catalogue number 14593, Redfern, Australia) and used as a positive control. All calpain inhibitor treatment compounds were dissolved in DMSO to create stock solutions and diluted in E3 zebrafish media to reach the final concentration. To confirm the effect of BLD-2673 on autophagic flux, co-treatment with 300 nM BLD-2736 and the autophagy inhibitor chloroquine diphosphate (3 mM dissolved in milliQ water, Sigma-Aldrich, catalogue number C6628, Castle Hill, NSW, Australia) was also performed. Vehicle treatment consisted of E3 medium containing DMSO.

### 2.3. Zebrafish Swimming Analysis

Examination of zebrafish movement in response to darkness was conducted using Viewpoint ZebraLab tracking software, as previously described [43]. In brief, 6 dpf zebrafish larvae were transferred to a 24-well plate and allowed to acclimate for 20 min. Larvae were then exposed to light within the ZebraLab for six min and then four minutes of darkness. The total distance travelled by larvae during the dark phase was calculated and statistically analyzed.

### 2.4. Flow Cytometric Analysis of Insoluble Ataxin-3

Whole zebrafish larvae were euthanized and dissociated into a cell solution using previously described methods [45]. Dissociated cells from pooled larvae samples were aliquoted and lysed using PBS containing 0.5% Triton-X, protease inhibitors and 1× Red Dot (nuclear stain), allowing enumeration of the number of detergent insoluble particles per nuclei. Samples were incubated on ice and protected from light until analysis. All samples underwent flow cytometric analysis within 30 min of the addition of lysis buffer.

Flow cytometry was performed using a Becton Dickson Biosciences LSR Fortessa analytical flow cytometer, running FACS DIVA software and maintained according to the manufacturer’s instructions (Becton Dickson, Franklin Lakes, NJ, USA). The fluorescence of EGFP-expressing cells was compared to a non-transgenic control samples. Nuclei were identified and quantified based on intensity of infrared fluorescence and particle size (forward scatter). The number of insoluble EGFP particles, indicating insoluble EGFP-fused ataxin-3 particles, were analyzed based on GFP fluorescent intensity and forward scatter [45,54]. The number of insoluble EGFP^+^ particles was expressed as a proportion of the number of nuclei present, analyzed as a ratio of insoluble EGFP particles per nuclei.

### 2.5. Western Blotting

To prepare protein lysates to allow immunoblot analysis of proteins expressed by the zebrafish larvae, six-day old zebrafish larvae were euthanized and homogenized in RIPA buffer (2 μL per larvae) containing protease inhibitors (Complete ULTRA Tablets, Roche, Sydney, Australia) using a manual dounce. Homogenates were centrifuged for 20 min at 4 °C and the supernatant was collected. Protein concentration was determined using a Pierce™ BCA Protein Assay Kit (Thermo Fisher Scientific, Brisbane, Australia). Equal amounts of protein were separated using SDS-PAGE and transferred to a PVDF (0.45 μm pore) or nitrocellulose membrane (0.2 μm pore) for immunoblot probing. To confirm the effect of calpain inhibition on ataxin-3 protein cleavage and autophagic activity, immunoblots were probed with rabbit anti-MJD (1:80,000; kind gift from H. Paulson), rabbit anti-LC3 (1:2500; Abcam, RRID: AB_881429, Cambridge, UK), or mouse anti-GAPDH (1:5000; Proteintech, RRID: AB_2107436, Rosemont, IL, USA). The immunoblots were probed with the appropriate secondary antibodies (Promega, Alexandria, Australia) and visualized using chemiluminescence (Supersignal detection kit, Pierce, Brisbane, Australia) using an ImageQuant LAS4000 imaging system or Licor Odyssey CLX fluorescent imaging system. The intensity of bands within the immunoblot were quantified by Image Studio Lite and the target protein expression level was determined by normalizing against a house keeping protein (GAPDH).

### 2.6. Data Analysis

Data analysis was performed using GraphPad Prism (version 9) software. Group comparisons were made using one-way analysis of variance (ANOVA) tests, followed by Tukey’s posthoc test to identify differences. A Shapiro–Wilk test was used to confirm normal distribution across examined data sets. All data is presented as fold change (relative to the vehicle treated EGFP ataxin-3 84Q larvae) ± standard error mean (SEM). Statistical significance is defined as *p* ≤ 0.05.

## 3. Results

### 3.1. Treatment with BLD-2736 Improves Swimming of SCA3 Zebrafish Larvae

**Figure 1:**
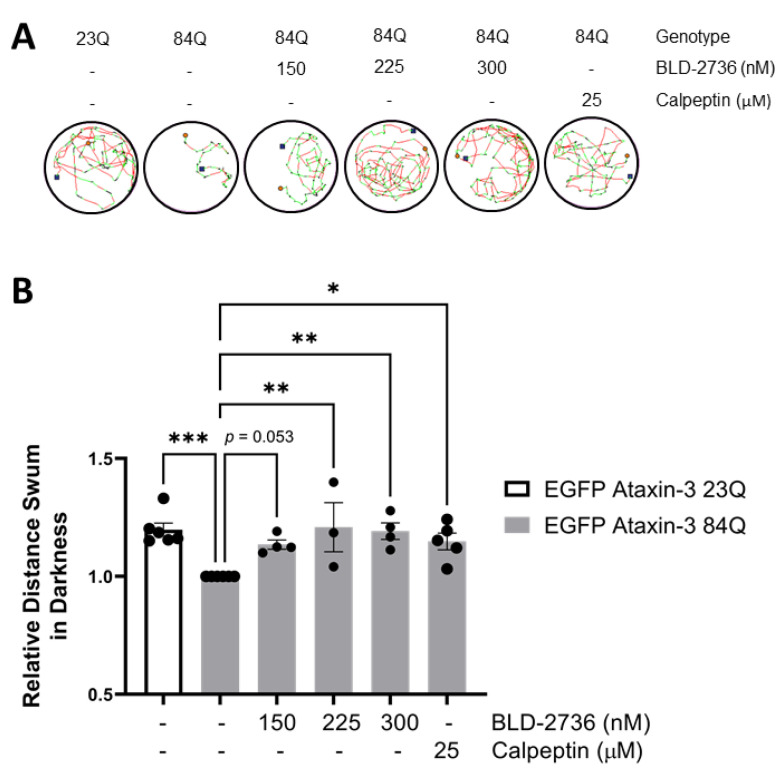
Treatment with BLD-2736 from 1 to 6 days post-fertilization improves SCA3 zebrafish swimming performance. (**A**) Representative trajectories displaying the distance swum during motor performance assays at 6 days post-fertilization. (**B**) Transgenic zebrafish expressing EGFP-fused human ataxin-3 with 84 polyglutamines swim shorter distances than zebrafish expressing EGFP-fused human ataxin-3 with 23 polyglutamines. Treatment with calpain inhibitors (BLD-2736 or calpeptin) improved the distance swum. All data are presented as mean ± SEM, *n* = 12–24 larvae were examined per group. * represents *p* < 0.05, ** represents *p* < 0.01, *** represents *p* < 0.001.

To examine whether treatment with the novel calpain inhibitor BLD-2736 had beneficial effects for the treatment of SCA3, we first tested the effect of BLD-2736 treatment on swimming performance in 6-day old SCA3 (EGFP ataxin-3 84Q) zebrafish larvae. We found that treatment with BLD-2736 from 1–6 dpf resulted in a dose dependent increase in the distance swum by the SCA3 zebrafish, with the optimal benefit at 225–300 nM (Figure 1A,B). As reported previously, treatment with calpeptin, also improved the distances swum by SCA3 zebrafish; however, requiring a much higher concentration (25 μM). One-way analysis of variance revealed a statistically significant difference across the examined groups (*p* = 0.0017). Post hoc comparisons revealed that SCA3 zebrafish swam a shorter distance when compared to zebrafish larvae expressing EGFP ataxin-3 with a short (23Q) polyQ repeat (*p* = 0.0007), as previously reported [43,51]. Furthermore, treatment with 225 nM BLD-2736, 300 nM BLD-2736, and 25 μM calpeptin each increased the total distance swum compared to vehicle treated SCA3 zebrafish larvae (*p* = 0.0035, *p* = 0.0033 and *p* = 0.0181, respectively). Whilst treatment with 150 nM BLD-2736 yielded an improvement in the mean relative distance swum, direct comparison of this dose with vehicle treatment did not reach statistical significance (*p* = 0.0531).

### 3.2. Treatment with BLD-2736 Decreases the Presence of Protein Aggregates and PolyQ Expanded Ataxin-3 in SCA3 Zebrafish

**Figure 2:**
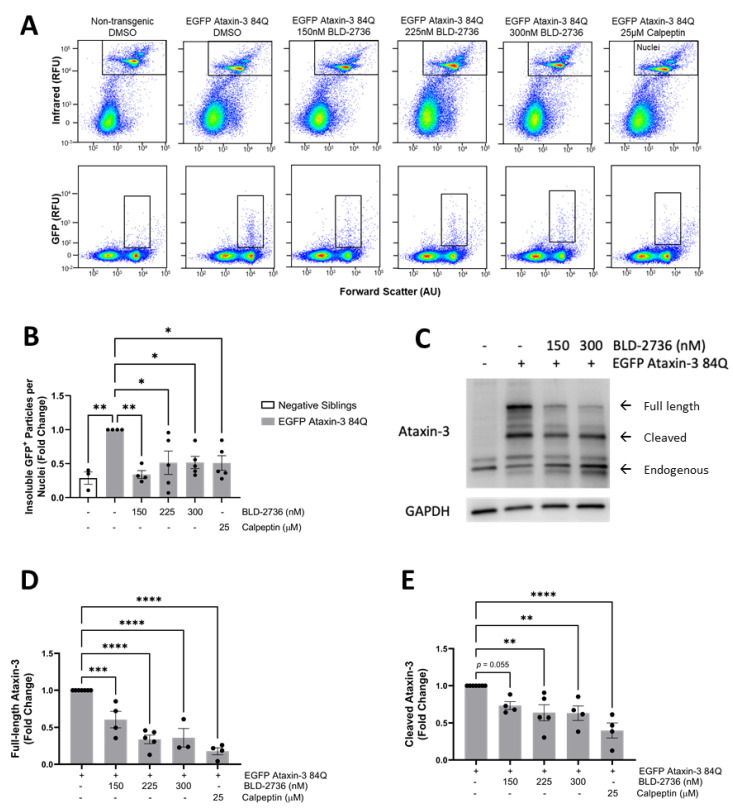
Treatment with BLD-2736 from 1 to 6 days post-fertilization reduces the presence of ataxin-3 protein species. (**A**) Representative scatterplots showing flow cytometric detection of detergent-insoluble EGFP-fused ataxin-3 particles following calpain inhibitor treatments. (**B**) The number of detergent-insoluble EGFP particles relative to nuclei was decreased following treatment with calpain inhibitors. (**C**) Representative western blot showing the presence of full length and cleaved ataxin-3 protein species. (**D**) Calpain inhibitor treatments were found to decrease the presence of full length ataxin-3 species in a dose-dependent manner. (**E**) Calpain inhibitor treatments also produced a decrease in the presence of cleaved ataxin-3 species, as detected by Western blotting. All data are presented as mean ± SEM. Each data point represents a single experimental replicate, where samples were pooled from *n* = 12–20 zebrafish larvae. * represents *p* < 0.05, ** represents *p* < 0.01, *** represents *p* < 0.001, **** represents *p* < 0.0001.

We have recently developed a methodology for detecting EGFP-fused human ataxin-3 protein aggregates in transgenic zebrafish larvae, using a modification of a previously published methodology called FloIT [45,54]. This methodology involves lysing homogenates of the euthanized zebrafish larvae with Triton-X and then using flow cytometry to quantify the number of Triton-X insoluble EGFP^+^ particles. Using this methodology, we previously reported that our EGFP ataxin-3 84Q zebrafish carried higher numbers of detergent insoluble EGFP^+^ particles than EGFP ataxin-3 23Q zebrafish or non-transgenic controls at both 2 dpf and 6 dpf. Applying this protocol to samples of EGFP ataxin 3 84Q zebrafish larvae treated with BLD-2736, calpeptin, or vehicle control from 1 dpf revealed that both BLD-2736 and calpeptin were effective at decreasing the number of protein aggregates relative to the number of nuclei at 6dpf (one way ANOVA: *p* = 0.0035, Figure 2A,B). Post hoc comparisons revealed that vehicle treated EGFP ataxin-3 84Q larvae displayed significantly more Triton-X insoluble particles compared to non-transgenic siblings (*p* = 0.0009). Interestingly, treatment with 150 nM of BLD produced the greatest decrease in insoluble particles when compared to the vehicle treatment (*p* = 0.0021), with 225 nM BLD, 300 nM BLD, and 25 μM calpeptin also revealing a decrease in the number of insoluble particles when compared to the vehicle treatment (*p* = 0.0173, *p* = 0.0183 and *p* = 0.0164, respectively).

In line with these findings, immunoblot analysis of protein lysates from SCA3 larvae treated with BLD-2736 or calpeptin from 1 to 6 dpf revealed the decreased presence of the ataxin-3 protein (Figure 2C, see Figure S1 for uncropped western blot images). Quantification revealed that full length ataxin-3 protein levels were decreased following treatment with both calpain inhibitor compounds (one way ANOVA *p* < 0.0001). Treatment with 150 nM, 225 nM, and 300 nM BLD-2736 was found to significantly decrease full length ataxin-3 levels when compared to the vehicle treatment (*p* = 0.0009, *p* < 0.0001, and *p* < 0.0001, respectively, Figure 2D). As previously reported [43], treatment with 25 μM calpeptin was also found to decrease expression of full-length ataxin-3, when compared to the vehicle treatment (*p* < 0.0001). Examination of cleaved ataxin-3 fragments also revealed a significant effect of calpain inhibitor treatments (*p* = 0.0002). Post hoc comparisons revealed a significant decrease in the presence of cleaved ataxin-3 species following treatment with 225 nM BLD-2736, 300 nM BLD-2736, and 25 μM calpeptin (*p* = 0.0042, *p* = 0.0064 and *p* < 0.0001, respectively, Figure 2E). Interestingly, treatment with 150 nM BLD-2736 reduced the mean relative amount of cleaved ataxin-3 species when compared to vehicle treated controls, however, this comparison failed to reach statistical significance (*p* = 0.0553).

### 3.3. Delayed BLD-2736 Treatment Can Remove Formed Ataxin-3 Aggregates

**Figure 3:**
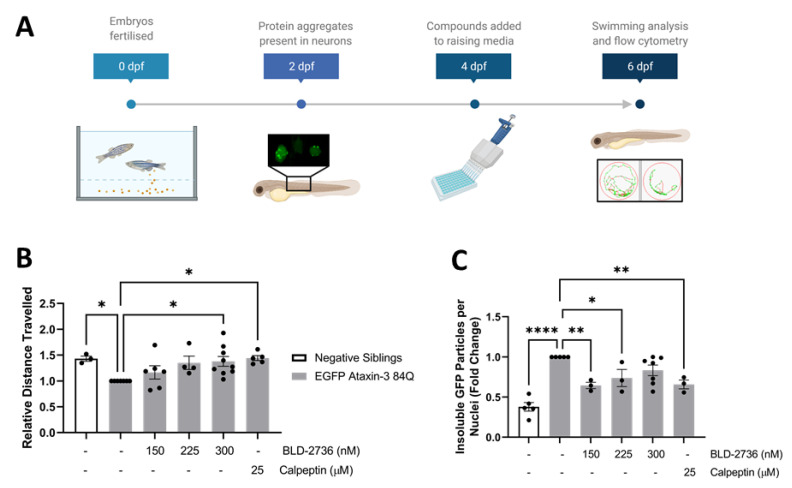
Delayed treatment with BLD-2736 or calpeptin produced a therapeutic benefit, despite administration after the onset of disease pathology. (**A**) Timeline displaying the onset of disease pathology at 2 dpf, treatment administration at 4 dpf, and examination of treatment efficacy at 6 dpf. (**B**) Treatment of SCA3 zebrafish from 4 to 6 dpf improved the distance swum when compared to vehicle treated SCA3 zebrafish. Swimming analysis was performed on *n* = 12*–*25 larvae per group. (**C**) Post-symptomatic treatment with calpain inhibitors produced a detectable decrease in the presence of detergent-insoluble EGFP-fused ataxin-3 particles. Each data point represents an experimental replicate, where *n* = 12*–*20 larvae were pooled together. All data are presented as mean ± SEM. * represents *p* < 0.05, ** represents *p* < 0.01, **** represents *p* < 0.0001.

To better understand the translational potential of calpain inhibitor treatments, we next aimed to confirm whether the administration of calpain inhibitors yielded a therapeutic benefit in SCA3 zebrafish through the removal of protein aggregates rather than prevention of aggregate formation. To examine whether the BLD-2736 treatment was able to aid the removal of protein aggregates that had already formed, we tested the effect of delaying the commencement of BLD-2736 treatment. We previously reported the presence of increased numbers of detergent insoluble particles, as detected by flow cytometry, in 2 dpf EGFP ataxin-3 84Q zebrafish larvae [45]. Thus, in this series of experiments we chose to commence BLD-2736 and calpeptin treatments at 4 dpf, after the onset of a detectable protein aggregate phenotype (Figure 3A). We found that 2 days of treatment (from 4 to 6 dpf) with 300 nM BLD and 25 μM calpeptin was sufficient to rescue swimming deficits in SCA3 zebrafish expressing EGFP-fused human ataxin-3 84Q (one way ANOVA: *p* < 0.0188, Figure 3B). Treatment with 300 nM BLD was found to improve the mean distance swum relative to the vehicle treatment (*p* = 0.0125), with 25 μM calpeptin also yielding improvements compared to the vehicle treatment (*p* = 0.0119).

Immediately upon completion of movement assays, zebrafish larvae were euthanized and dissociated for flow cytometric analysis of detergent insoluble EGFP-fused ataxin-3 particles. Here, we report that treatment with calpain inhibitors altered the number of detergent insoluble EGFP ataxin-3 particles, as detected by FloIT (one way ANOVA: *p* < 0.0001, Figure 3C). Post hoc comparisons revealed that treatment with 150 nM and 225 nM BLD-2736 from 4 to 6 dpf resulted in a decreased presence of EGFP ataxin-3 detergent insoluble ataxin-3 particles when compared to the vehicle treatment (*p* = 0.0054 and *p* = 0.0473, respectively). Similarly, delayed treatment with calpeptin also reduced the number of detergent insoluble EGFP^+^ particles detected by flow cytometry, when compared to the vehicle treated EGFP ataxin-3 84Q larvae (*p* = 0.0074). Interestingly, delayed treatment with 300 nM BLD-2736 did not yield a statistically significant decrease in detergent insoluble particles when compared to the vehicle treatment (*p* = 0.1473).

### 3.4. Treatment with BLD-2736 Increased Synthesis of LC3II and Autophagic Activity

**Figure 4:**
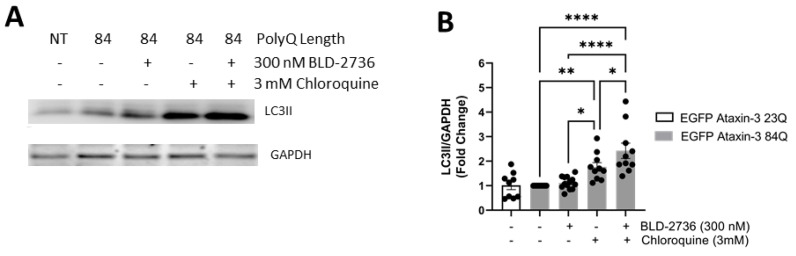
Treatment with BLD-2736 increased the activity of the autophagy protein quality control pathway. (**A**) Immunoblotting of LC3II revealed changes in the levels of LC3II relative to GAPDH across treatment groups. (**B**) Quantitative analysis of the ratio of LC3II to GAPDH was found to be significantly increased in larvae treated with 300 nM BLD-2736 when compared to larvae treated with the autophagy inhibitor chloroquine and 300 nM BLD-2736, indicating increased synthesis of LC3II and induction of autophagy. Protein lysates were produced from pooled samples of *n* = 12*–*20 zebrafish larvae. Each data point represents an independent experimental replicate. All data are presented as mean ± SEM. * represents *p* < 0.05, ** represents *p* < 0.01, **** represents *p* < 0.0001.

In view of our finding of decreased detergent insoluble EGFP ataxin-3 in our SCA3 zebrafish larvae following treatment with BLD-2736, we sought to examine whether BLD-2736 treatment was inducing activity of the autophagy protein quality control pathway. To confirm that treatment with BLD-2736 was altering the activity of the autophagy pathway, we examined the level of LC3II relative to GAPDH in lysates from EGFP ataxin-3 23Q larvae and EGFP ataxin-3 84Q larvae treated with BLD-2736 (300 nM), the autophagy inhibitor chloroquine (3 mM), and larvae co-treated with both BLD-2736 and chloroquine (Figure 4A, see Figure S2 for uncropped western blot images). We detected a difference in LC3II/GAPDH levels across groups, which reached statistical significance (*p* < 0.0001, Figure 4B). Post hoc comparisons revealed that samples from larvae treated with BLD-2736 did not have increased LC3II/GAPDH compared to those from larvae treated with the vehicle (*p* = 0.981). This finding does not preclude that BLD-2736 is an autophagy inducer, because at basal conditions, without cotreatment with a lysosomal inhibitor, both autophagosome formation and degradation may simultaneously be increased [55]. EGFP ataxin-3 84Q larvae treated with chloroquine did have statistically higher LC3II/GAPDH compared to those treated with vehicle alone, suggesting that autophagic flux occurs at baseline in EGFP-ataxin-3 84Q larvae (*p* = 0.011). However, larvae co-treated with BLD-2736 and chloroquine together had higher levels of LC3II/GAPDH than larvae treated with chloroquine alone (*p* = 0.0265) and BLD-2736 alone (*p* < 0.0001). These findings suggest that treatment with 300 nM BLD-2736 increased both the synthesis and degradation of autophagosomes, thus increasing autophagic flux in agreement with the accepted guidelines for demonstrating increased autophagy induction [55].

## 4. Discussion

Here, we report the therapeutic potential of BLD-2736, a novel inhibitor of calpain-1, 2, and 9 and cathepsin K, for the treatment of SCA3, following the detection of beneficial effects from treatment of SCA3 zebrafish larvae. We found that treatment with BLD-2736 from 1 to 6 dpf improved the swimming of the SCA3 zebrafish larvae. Immunoblotting of protein lysates obtained from SCA3 larvae treated from 1 to 6 days post-fertilization also revealed a dose-dependent decrease in the amount of cleaved ataxin-3 species. These findings are consistent with previous reports that ataxin-3 is cleaved by calpain proteases [37,38,56], and that treatment with calpain inhibitors can decrease the presence of cleavage fragments [38,41,42].

To extend these findings further, we applied our recently developed flow cytometry methodology for quantifying the presence of protein aggregates within an in vivo model [45]. We found that treatment with BLD-2736 resulted in decreased presence of detergent insoluble EGFP-fused ataxin-3 protein aggregates in SCA3 zebrafish, when compared to vehicle treated controls. This finding is in line with our recent report of the capacity of treatment with calpeptin, a calpain-1 and 2 inhibitor, to decrease the presence of protein aggregates in vivo [45].

The flow cytometry approach employed in the present study offers many advantages over more traditional microscopy approaches. First, flow cytometric analysis of inclusions and trafficking (FloIT) is a relatively rapid, high-throughput approach which, can provide quantification of EGFP^+^ particles within minutes of flow cytometric analysis of cells from dissociated larvae. Second, the use of Triton-X 100 lysis allows rapid identification of detergent-insoluble, and thus likely to be toxic, protein species. Analysis of insoluble protein species is likely more relevant to the understanding of proteinopathy diseases, rather than quantification of proteins species via traditional microscopy methods, which cannot differentiate soluble from toxic, insoluble protein species. Furthermore, we found our flow cytometry assay to be more sensitive at detecting neuroprotective doses of BLD-2736, finding a statistically significant reduction in insoluble EGFP-fused ataxin-3 particles in larvae treated from 1 to 6 dpf, even with 150 nM BLD-2736, the lowest dose examined. This study demonstrates the vast potential for using flow cytometry as a method for quantifying levels of protein aggregation, even within an in vivo model [45].

Interestingly, our immunoblot analysis also revealed a dose-dependent decrease in full length EGFP-fused ataxin-3 protein following treatment with BLD-2736. This reduction in detergent insoluble ataxin-3 aggregates and clearance of full length EGFP ataxin-3 protein led us to hypothesize that the novel calpain inhibitor BLD-2736 may induce autophagic activity. Given that in this model EGFP-fused ataxin-3 is expressed exclusively in neurons, we can conclude that despite BLD-2736 treating the whole organism, our findings demonstrate that BLD-2736 is capable of inhibiting calpains and inducing autophagy in neurons.

We hypothesize that calpain inhibitor treatments, such as BLD-2736 or calpeptin, may increase the activity of the autophagy protein quality control pathway through inhibiting aberrant cleavage of key autophagy-related proteins. To examine this, we performed immunoblot analysis on protein lysates generated from larvae treated with BLD-2736, the autophagy inhibitor chloroquine or combined treatment with both BLD-2736 and chloroquine. Indeed, we can report that co-treatment with BLD-2736 and chloroquine produced an increase in LC3II/GAPDH compared to treatment with BLD-2736 or chloroquine alone, indicating increased synthesis of LC3II following treatment with BLD-2736 [55]. These findings align with existing reports, from our team and others, that calpain inhibition, via treatment with exogenous calpain inhibitor compounds or overexpression of calpastatin, can activate clearance of accumulated proteins via increasing the activity of the autophagy protein quality control pathway [43,46,47,57,58]. Whilst it may be questioned whether the treatment with BLD-2736 may have instead decreased full-length ataxin-3 through effects on the expression of EGFP-ataxin-3 via an effect on transcription, we previously demonstrated that treatment with calpeptin decreased the presence of full length human ataxin-3 protein, without altering the levels of human ataxin-3 mRNA, and that this effect was prevented by cotreatment with the autophagy inhibitor, chloroquine [43].

One mechanism through which calpain inhibitors have been proposed to induce autophagy involves prevention of the cleavage of important autophagy-related proteins; modulating the activation of these proteins [46,50,57,58,59,60]. Proteins important for autophagosome formation, such as Atg5 and Beclin-1, are known substrates of calpains [50,60]. Cleavage of Atg5 and Beclin-1 causes inactivation of these proteins, consequently inhibiting autophagic activity [50,61]. Under normal functioning, calpain-1 cleavage of Atg5 functions to tightly control basal autophagic activity, with cleavage of Atg5 downregulating the formation of Atg5-Atg12 complexes and inhibiting autophagy induction [59]. Furthermore, Beclin-1 has previously been reported to be downregulated in MJD patient fibroblasts and mouse models of MJD, causing impairments in autophagosome formation [62,63]. Aberrant cleavage of Atg5 and Beclin-1 may result in chronic inactivation of these proteins, consequently producing a deficit in the ability of autophagy pathways to degrade aggregation-prone proteins [64]. Thus, the inhibition of calpain proteases may enhance activity of the autophagy protein quality control pathway by decreasing proteolytic cleavage and inactivation of Atg5 and Beclin-1, leading to the observed increase in LC3II synthesis. Further research is required to confirm the precise mechanism by which inhibition of calpains can trigger increased autophagic activity and LC3II synthesis.

In addition to the role of calpains in modulating Beclin-1 function, there is also evidence to suggest that the polyQ stretch within wild type ataxin-3 protein may interact and bind with Beclin-1, initiating autophagy and preventing degradation of Beclin-1 via the ubiquitin-proteosome system [65]. It is hypothesized that pathological expansion of the polyQ stretch within the ataxin-3 protein may hinder functional interactions with Beclin-1, causing defects in basal autophagy [65]. It is plausible that calpain inhibitor compounds may alter the stability of Beclin-1 and rectify the functional interaction between ataxin-3 and Beclin-1, restoring basal autophagy. The specific mechanism through which calpain inhibitor treatments increase autophagic activity in our transgenic zebrafish model of SCA3 is yet to be fully elucidated.

These findings indicate that treatment with BLD-2736 may produce beneficial effects in SCA3 zebrafish via either decreasing cleavage of the ataxin-3 protein and/or increased autophagic clearance of any neurotoxic ataxin-3 protein aggregates. Decreased ataxin-3 cleavage may result in decreased formation of neurotoxic ataxin-3 protein aggregates, through decreased presence of the lone aggregation prone polyglutamine containing peptide. Furthermore, aberrant cleavage can perturb the function of a range of essential proteins and increase the presence of smaller fragments that can readily accumulate and form neurotoxic protein aggregates [24,38,41].

Existing works by Koch et al. [38] and Weber et al. [40] highlighted that increased activity of calpains in SCA3 patient cells and that calpain overactivity likely contributes to SCA3 pathogenesis. Nevertheless, further investigation into the separate contributions of decreased calpain activity and increased autophagy induction in producing this therapeutic benefit is still required.

To further explore the translational potential of BLD-2736, we also tested the efficacy of delaying treatment administration until after the onset of disease phenotypes in the zebrafish. We previously reported that detergent insoluble ataxin-3 aggregates can be detected in SCA3 zebrafish larvae as early as 2 dpf [45]. As such, we wanted to explore the potential efficacy of BLD-2736 if administered after the onset of a protein aggregation phenotype. Treatment of SCA3 zebrafish from 4 to 6 days post-fertilization still yielded significant improvements in the total distance swum in response to darkness, and again produced a significant decrease in insoluble EGFP-fused ataxin-3 particles when compared to vehicle treated controls. Excitingly, this finding suggests that treatment with BLD-2736 may be beneficial after the onset of ataxin-3 protein aggregates. This finding has important implications for SCA3 treatment, as it indicates its potential as a treatment after the initiation of disease symptoms. In light of these findings, BLD-2736 warrants further investigation in proteinopathy diseases, both with and without a genetic basis, wherein patients may benefit from treatments found to be effective after the onset of disease pathogenesis.

In the present study, we compared the efficacy of a novel calpain inhibitor, BLD-2736, to calpeptin, a compound previously shown to produce neuroprotection in the same transgenic zebrafish model of SCA3 [43]. Interestingly, we found BLD-2736 was able to produce beneficial effects on SCA3 zebrafish at a much lower dose than calpeptin (efficacy found at nanomolar concentrations for BLD-2736, whilst calpeptin produces benefits at doses in the micromolar range). This suggests that BLD-2736 may have an increased potency over calpeptin. Furthermore, BLD-2736 is an inhibitor of calpain-1, calpain-2, calpain-9, and cathepsin K, whilst calpeptin is known to inhibit calpain-1, calpain-2, cathepsin L, and cathepsin K [66]. Calpain-1 and calpain-2 are ubiquitously expressed proteolytic enzymes that have been implicated in many neurodegenerative diseases and neurological disorders [67,68]. In contrast, calpain-9 has been found to be predominately expressed in the stomach [69], and altered expression of calpain-9 has been associated with poor prognosis in cancer patients and increased apoptosis [70,71]. Interestingly, treatment with high dose BLD-2736 (300 nM) was consistently found to yield the greatest improvement in zebrafish swimming, following both pre-symptomatic and post-symptomatic treatment administration. In contrast, low dose BLD-2736 (150 nM) was found to yield the greatest reduction in detergent-insoluble EGFP-fused ataxin-3 particles, as detected by flow cytometry. We hypothesize that our flow cytometric analysis of insoluble ataxin-3 species may be a more sensitive method of detecting beneficial effects, as we observed statistically significant reductions in insoluble ataxin-3 particles at all examined doses of BLD-2736 in SCA3 zebrafish larvae treated from 1 to 6 dpf. These findings were also supported by our western blotting results, which provided evidence of decreased full length ataxin-3 at all examined doses of BLD-2736. Furthermore, we found that 150 nM of BLD-2736 was capable of improving the distance swum and that this effect neared statistical significance (*p* = 0.053). Collectively, we suggest that BLD-2736 is a promising therapeutic that ameliorates multiple disease phenotypes characteristic of SCA3.

Here, we report for the first time that a novel therapeutic, the calpain inhibitor BLD-2736, can prevent development of motor impairment, decrease the presence of ataxin-3 cleavage fragments and protein aggregates, and induce autophagy activity. Our findings indicate that calpain inhibitor compounds may prove efficacious, not just at early disease stages but also after the formation of protein aggregate pathology. Indeed, our data suggest that treatment with calpeptin or BLD-2736 following the formation of insoluble ataxin-3 aggregates in SCA3 zebrafish can still yield therapeutic benefits. Nevertheless, further preclinical studies are required to determine at which stages of disease progression whereby treatment is no longer effective. Finally, we propose that these findings, of the beneficial effects of treatment with BLD-2736, have potential, not just for the treatment of SCA3, but for a broad range of proteinopathy diseases where the induction of autophagy has been demonstrated to have beneficial effects [45,48,49,59].

## Data Availability

The datasets used in the current study are available from the corresponding author on reasonable request.

## Supplementary Materials

The following are available online at https://www.mdpi.com/article/10.3390/cells10102592/s1, Figure S1: Uncropped Western blot images from Figure 2C, Figure S2: Uncropped Western blot images from Figure 4A.
