# Supplementary material for: A Novel Calpain Inhibitor Compound Has Protective Effects on a Zebrafish Model of Spinocerebellar Ataxia Type 3
Source: Cells. 2021 Sep 29;10(10):2592. doi: 10.3390/cells10102592 (PMC8533844; doi:10.3390/cells10102592)
Supplement: Supplementary file 1 [file cells-10-02592-s001.zip › cells-1313263-supplementary.pdf]

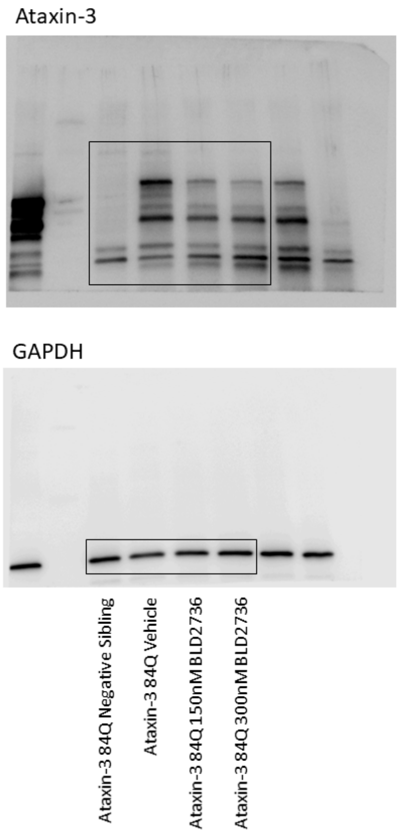

Supplementary Figure S1. Uncropped Western blot images from Figure 2C.

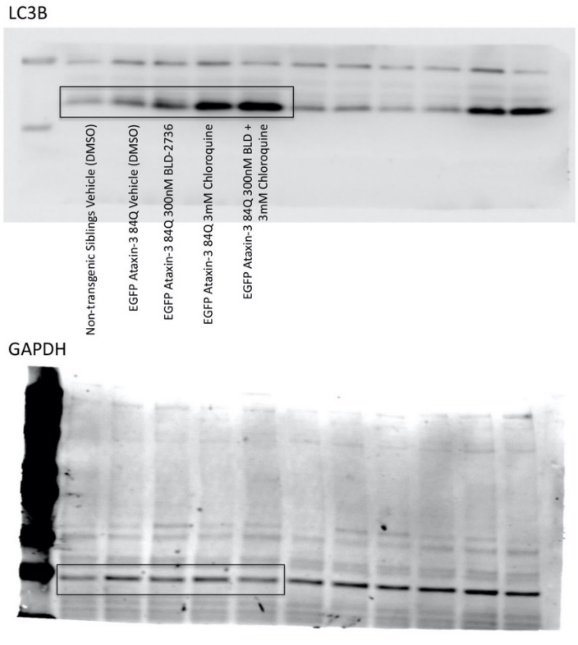

Supplementary Figure S2. Uncropped Western blot images from Figure 4A.
